# The pre/parasubiculum: a hippocampal hub for scene-based cognition?

**DOI:** 10.1016/j.cobeha.2017.06.001

**Published:** 2017-10

**Authors:** Marshall A Dalton, Eleanor A Maguire

**Affiliations:** Wellcome Trust Centre for Neuroimaging, Institute of Neurology, University College London, 12 Queen Square, London WC1N 3BG, UK

## Abstract

Internal representations of the world in the form of spatially coherent scenes have been linked with cognitive functions including episodic memory, navigation and imagining the future. In human neuroimaging studies, a specific hippocampal subregion, the pre/parasubiculum, is consistently engaged during scene-based cognition. Here we review recent evidence to consider why this might be the case. We note that the pre/parasubiculum is a primary target of the parieto-medial temporal processing pathway, it receives integrated information from foveal and peripheral visual inputs and it is contiguous with the retrosplenial cortex. We discuss why these factors might indicate that the pre/parasubiculum has privileged access to holistic representations of the environment and could be neuroanatomically determined to preferentially process scenes.

## Highlights

• We consider internal representations of the world in the form of scenes.
• The anterior medial hippocampus is implicated in scene-based cognition.
• This region contains the pre/parasubiculum.
• The pre/parasubiculum is a primary target of a major visuospatial processing system.
• The pre/parasubiculum may be the hippocampal hub of the scene processing network.

**Current Opinion in Behavioral Sciences** 2017, **17**:34–40

This review comes from a themed issue on **Memory in time and space**

Edited by **Lila Davachi** and **Neil Burgess**

For a complete overview see the Issue and the Editorial

Available online 15th June 2017

**http://dx.doi.org/10.1016/j.cobeha.2017.06.001**

2352-1546/© 2017 The Authors. Published by Elsevier Ltd. This is an open access article under the CC BY license (http://creativecommons.org/licenses/by/4.0/).

The precise role of the hippocampus in cognition remains enigmatic. Traditionally associated with episodic memory [1], neuroimaging and neuropsychological studies have consistently implicated the human hippocampus in a range of other functions including the imagination of fictive and future experiences [2, 3], navigation [4•, 5], complex spatial perception [6, 7, 8] and decision-making [9, 10]. Notably, each of these functions seems to involve either recalling or creating an internal representation of the world which is couched within the visuospatial framework of a ‘scene’. Here, we define a scene as a naturalistic three dimensional space which one could potentially step into and operate within, viewed from a first person perspective and populated by objects. These observations led to the scene construction theory, which posits that one function of the hippocampus is to construct internal representations of scenes in the service of memory, navigation, imagination, decision-making and a host of other functions [11^••^]. Recent investigations have further refined our understanding of hippocampal involvement in scene-based cognition. Specifically, a portion of the anterior medial hippocampus is consistently engaged by tasks involving scenes [11^••^], although it is not yet clear why a specific subregion of the hippocampus would be preferentially recruited in this manner.

Here we review the extant evidence, drawing largely from advances in the understanding of visuospatial processing pathways. We propose that the anterior medial portion of the hippocampus represents an important hub of an extended network that underlies scene-related cognition, and we generate specific hypotheses concerning the functional contributions of hippocampal subfields.

## What part of the human anterior medial hippocampus is preferentially engaged by scenes?

**Figure 1:**
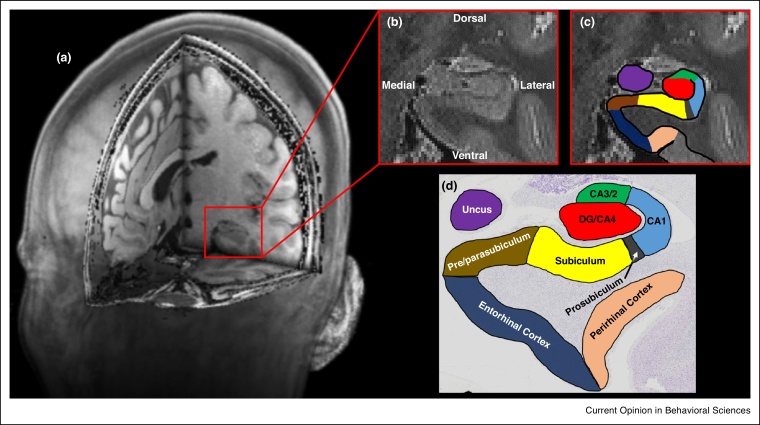
The location of the pre/parasubiculum. **(a)** A T1-weighted structural MRI scan presented in 3D with a block removed to reveal the location of the medial temporal lobe (red square). **(b)** A T2-weighted structural MRI scan showing magnification of the area encompassed within the red square in (a) showing the hippocampus in the coronal plane. **(c)** The same image presented in (b) overlaid with the approximate location of hippocampal subregions. **(d)** For comparative purposes, a histologically stained coronal section of the hippocampus overlaid with the approximate location of hippocampal subregions. Note the location of the pre/parasubiculum (brown) on the medial most extent of the hippocampus.

In a recent review, Zeidman and Maguire [11^••^] presented evidence from neuroimaging studies that showed the human anterior medial hippocampus is consistently recruited during recall, imagination and perception of scenes [2, 3, 12••, 13, 14]. The location of the activated voxels in the medial-most portion of the hippocampus aligns with the location of the presubiculum and parasubiculum [15^••^, replicated in Ref. 16] (Figure 1). Neuroanatomically, the presubiculum and parasubiculum are located medial to the subiculum and can be differentiated from it by specific structural characteristics [17, 18•, 19]. We know from rodent and non-human primate studies that the presubiculum and parasubiculum contain an abundance of grid, border and head direction cells [20, 21, 22, 23] each of which have been implicated in different aspects of spatial processing. Moreover, a computational model has shown how the interactions of these different cell types could theoretically give rise to mental imagery of a spatial scene [24, 25]. Taken together, these lines of evidence indicate that the presubiculum and parasubiculum are functionally associated with spatial, and possibly scene-based, cognition across mammalian species.

Considering humans, while advances in neuroimaging technology now permit a more detailed investigation of hippocampal subregions, the presubiculum and parasubiculum are nevertheless under-explored. The majority of human studies include the presubiculum and parasubiculum in a broader subiculum region of interest [26, 27] reflecting the technical difficulties associated with distinguishing these regions on MRI [28], but thereby missing the opportunity to investigate the specific functions of these structures. The few studies that have investigated their functional contributions to scene-based cognition have had to consider the two areas together in a combined region of interest because of limitations in the spatial resolution of most neuroimaging techniques. For expedience, we mirror this combined approach and refer to the ‘pre/parasubiculum’ hereafter as a single entity. Neuroimaging evidence supports the idea that the pre/parasubiculum is preferentially recruited during the construction of spatially coherent scenes [12••, 15••] and scene perception [12••, 16, 29]. Given these preliminary, but consistent, findings and the non-human and computational model observations noted above, it is now timely to consider why the pre/parasubiculum may be preferentially involved in processing spatially coherent scenes.

## Connectivity of the pre/parasubiculum

Visuospatial information is initially processed through an occipito-parietal network which stems from early visual cortical areas and projects to posterior regions of the parietal cortex [30, 31]. This dorsal visual processing stream has historically been implicated in aspects of spatial cognition [32, 33] but, more recently, three distinct pathways emerging from this dorsal stream have been characterised: a parieto-prefrontal pathway, a parieto-premotor pathway and a parieto-medial temporal pathway [34^••^]. The pre/parasubiculum is a primary hippocampal target of the parieto-medial temporal pathway. Here, we distil information relating to this pathway from the excellent review of Kravitz *et al*. [34^••^]. While patterns of connectivity have predominantly been characterised in non-human primates, evidence for the functional connectivity of homologous regions in the human brain support this framework [35, 36, 37].

**Figure 2:**
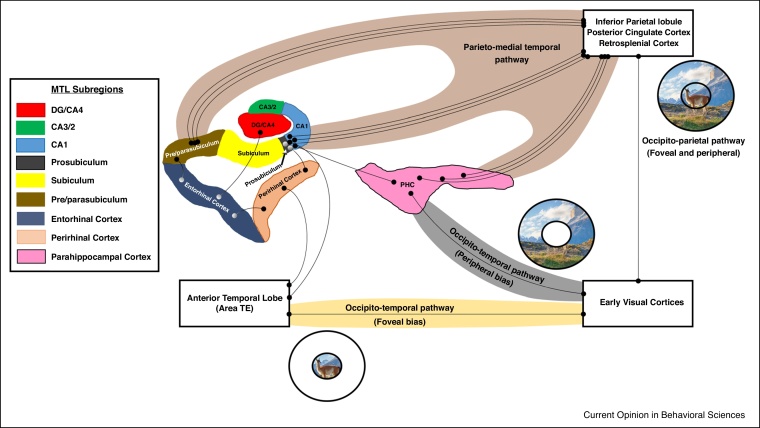
Visual processing pathways into the hippocampus. This schematic diagram represents the major inputs into the hippocampus through the dorsal parieto-medial temporal visuospatial processing pathway and the ventral occipito-temporal visual processing pathway. Note the preferential connectivity of the pre/parasubiculum with regions of the dorsal pathway (light brown background) while the prosubiculum/CA1 region has a more distributed pattern of connectivity directly from the dorsal pathway and indirectly through portions of the ventral pathway which display foveal (ivory background) and peripheral (grey background) biases. For simplicity, the PHC is presented to the right of the hippocampus but in fact this region is located in a more posterior region of the medial temporal lobe.

The parieto-medial temporal pathway stems from the caudal inferior parietal lobule (cIPL). It channels visuospatial information from parietal regions to specific regions of the medial temporal lobes. The cIPL sends direct projections to the posterior cingulate cortex (PCC), retrosplenial cortex (RSC) and parahippocampal cortex (PHC). Each of these regions is a key node of the parieto-medial temporal pathway and primarily implicated in visuospatial processing [38, 39, 40, 41, 42]. Importantly, the cIPL, PCC and RSC each send direct projections to the pre/parasubiculum giving it privileged access to this visuospatial information (Figure 2).

Taking this into account, we start to gain traction on the question of why the pre/parasubiculum may be preferentially involved in scene-based cognition. Considering it is a primary target of the parieto-medial temporal pathway, we propose that the pre/parasubiculum is the hippocampal hub of a broader scene processing network. However, it is not the only hippocampal target of this pathway. The cIPL, RSC and PCC also send projections to the prosubiculum/CA1 portions of the hippocampus which are not consistently engaged in neuroimaging investigations involving scenes. We will return to this point shortly.

## Foveal and peripheral processing streams

The dorsally-located parieto-medial temporal pathway is not the only means through which visual information reaches the medial temporal lobe. A recent examination of the occipito-temporal ventral visual stream, traditionally associated with object processing, revealed six separate pathways stemming from the core occipito-temporal system [43^••^]. In relation to the medial temporal lobe, separate projections from the occipito-temporal stream innervate the perirhinal cortex (PRC) and PHC and, importantly, these projections display neuroanatomically-determined biases in foveal and peripheral retinotopic processing. In brief, well-characterised retinotopic maps in early visual cortices show that different regions of the early visual cortices process foveal and peripheral information [44]. Foveal and peripheral processing areas have differential patterns of projection along the occipito-temporal stream [45, 46, 47, 48]. This means that retinotopic biases inherent to early visual processing regions may be propagated to higher order visual areas.

The PRC receives direct and indirect input from area TE [49, 50] in the anterior portion of the inferior temporal lobe, which displays strong foveal processing [43••, 47] (Figure 2). TE also projects directly to the prosubiculum/CA1 [47, 51]. Considering its connectivity with regions displaying a foveal bias, the consistently observed association of the PRC with object processing makes intuitive sense. From birth, we primarily focus our fovea on objects of interest in the environment. In essence, it is possible that the PRC either develops to become, or is evolutionarily conserved to be, an object processing area by virtue of its connectivity with regions of the occipito-temporal visual stream that display a strong foveal bias. The PRC propagates information to the prosubiculum/CA1 regions of the hippocampus [52] and also to the entorhinal cortex (ENT) [53]. These areas, therefore, may receive detailed foveal/object information through connectivity with the PRC. The ENT, in turn, has complex interactions with the pre/parasubiculum [54] and also projects to the dentate gyrus (DG) of the hippocampus [55].

In contrast, the PHC receives direct input from early visual area V4 which has a strong peripheral processing bias [48]. This may account for its putative role in processing spatial information. In essence, peripheral vision takes in information from the environment which surrounds our foveal focal point. While acknowledging that the PHC is implicated in a broad range of cognitive processes relating to topographic and spatial representations, neuronal activity in the PHC shows a peripheral bias [46], is known to be modulated by changes to stimuli in the periphery [56], shows a preference for images that include the scene background [57] and reflects spatial rather than categorical or contextual elements of real world scenes [40, 58]. These observations suggest that the PHC is involved in processing peripheral elements of space. The PHC transmits information to the prosubiculum/CA1 [43^••^], suggesting this region may also receive spatial information via this connection.

In relation to the dorsal occipito-parietal processing stream, while some studies have observed peripheral processing biases in regions of the parieto-occipital sulcus [59], the occipito-parietal stream is generally considered to integrate information from both foveal and peripheral visual fields equally [43••, 60]. This suggests that the downstream parieto-medial temporal pathway may process integrated foveal and peripheral information also. Concordant with this, the RSC is implicated in processing stable objects in the environment [42] but also displays a peripheral bias [46], and expresses a preference for images that include the scene background [57]. We speculate that regions of the parieto-medial temporal pathway project integrated holistic scene information directly to the pre/parasubiculum.

## The retrosplenial cortex: a posterior extension of the pre/parasubiculum?

**Figure 3:**
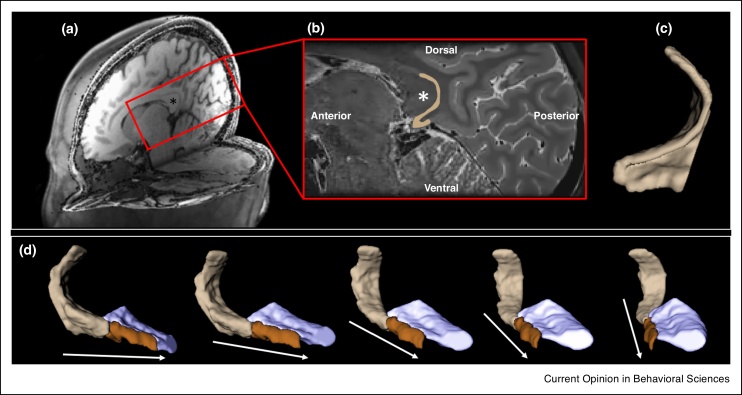
The pre/parasubiculum-retrosplenial cortex continuum. **(a)** A T1-weighted structural MRI scan presented in 3D with the left hemisphere removed to reveal a medial portion of the right hemisphere. **(b)** In this T2-weighted structural MRI scan sagittal section, the region encompassed within the red square in (a) is magnified. Note the thin band of the retrosplenial cortex (highlighted beige) hugging the ventral and posterior portion of the corpus callosum [* in both (a) and (b)]. **(c)** A 3D model of the retrosplenial cortex presented in the same orientation as the sagittal section presented in (b). **(d)** A 3D model of the retrosplenial cortex (beige), pre/parasubiculum (brown) and the posterior hippocampus (mauve) inclusive of all other hippocampal subregions. On the far left, the model is viewed from a medial perspective. Each consecutive image to the right represents an incremental rotation in a clockwise direction. Arrows indicate anterior. Note the contiguity between the posterior portion of the pre/parasubiculum and anterior portion of the retrosplenial cortex.

As noted above, the RSC is a key node of the parieto-medial temporal pathway and has been consistently implicated in visuospatial processing [39, 41, 42, 61]. Therefore, an additional point of interest is the pre/parasubiculum’s relationship with the RSC (Figure 3). It is easy to get the impression that the RSC is anatomically separate from but functionally related to the hippocampus. However, at the anatomical level, it can be argued that the RSC is actually an extension of the posterior hippocampus. Indeed some have referred to the RSC as part of the hippocampal formation [62]. In the adult mammalian brain, the RSC is anatomically contiguous with the posterior extent of the pre/parasubiculum.

When visualised using 3D modelling, the anatomical continuum between these structures is clear (Figure 3d). However, during primate foetal development the pre/parasubiculum and RSC initially develop separately. During the third trimester, the ventral/anterior most portion of the RSC merges with the dorsal/posterior most portion of the pre/parasubiculum [63] resulting in a gradual transition between these regions thereafter. Despite this anatomical contiguity and the proposal that these regions may constitute an anatomical-functional unit [62, 64], it should be noted that the RSC and pre/parasubiculum nevertheless express different patterns of connectivity [63]. The RSC has been postulated to translate between person-centred egocentric and world-centred allocentric reference frames [24, 25]. Whether the anatomical contiguity of the RSC and the pre/parasubiculum has functional significance for this process remains an open question. Considering the direct projections between the RSC and the pre/parasubiculum outlined above, it makes intuitive sense that these regions are functionally linked.

## Conclusions

Here we considered current evidence in order to explain why the pre/parasubiculum may be specifically involved in scene-based cognitive processing. We suggest that the pre/parasubiculum may be the hippocampal hub of an extended scene processing network which not only supports our ability to model the world during perception, but also to mentally construct internal scenes during episodic memory recall and imagination. As a primary target of the parieto-medial temporal visuospatial processing pathway, the pre/parasubiculum may have privileged access to holistic representations of the environment and be neuroanatomically determined to preferentially process scenes.

There are, however, some potential issues to bear in mind. For example, the pre/parasubiculum and prosubiculum/CA1 have similar patterns of input from the parieto-medial temporal pathway. However, while the pre/parasubiculum is consistently observed during functional MRI investigations of scene processing, the prosubiculum/CA1 is usually not. Why this is the case remains unclear. However, given its more specific connectivity with nodes of the parieto-medial temporal pathway, it is possible that the pre/parasubiculum has a specific role in the holistic representation of scenes. In contrast, the prosubiculum/CA1 displays a more distributed pattern of connectivity potentially requiring a division of labour between foveal/object information from the PRC, peripheral/spatial information from the PHC and more holistic scene information from the parieto-medial temporal pathway.

The framework proposed here suggests predictions for hippocampal subregion contributions to scene construction when compared with other types of complex visuospatial representations. We predict that internal representations of scenes within a naturalistic 3D framework will preferentially recruit the pre/parasubiculum and regions of the parieto-medial temporal visuospatial pathway including the RSC, PCC and PHC. In addition, considering its connectivity with object/foveal processing regions via the PRC, we hypothesise that areas within the lateral hippocampus corresponding with the location of the prosubiculum/CA1 will be specifically recruited during the internal representation of objects.

In closing, we acknowledge that the connectivity of the regions discussed here is much more complex than we had space to present and, as illustrated by Kravitz *et al*. [43^••^], by no means involves purely feedforward systems. Our goal instead was to propose a simple rationale for why the pre/parasubiculum is implicated in the scene-based cognition that seems to be so central to our mental life [11^••^]. It should be noted that our rationale does not preclude the existence of other mechanisms within the pre/parasubiculum, and future work is required to validate or refute this framework. Moreover, going forward it will be important to establish the separate contributions of the presubiculum and parasubiculum along with other subregions of the hippocampus, and to investigate potential top-down influences from the prefrontal cortex on the perception, construction and memory of scenes.

## References and recommended reading

Papers of particular interest, published within the period of review, have been highlighted as:

• of special interest
•• of outstanding interest

## Conflict of interest

Nothing declared.
